# The duodenal microbiome is altered in small intestinal bacterial overgrowth

**DOI:** 10.1371/journal.pone.0234906

**Published:** 2020-07-09

**Authors:** Gabriela Leite, Walter Morales, Stacy Weitsman, Shreya Celly, Gonzalo Parodi, Ruchi Mathur, Gillian M. Barlow, Rashin Sedighi, Maria Jesus Villanueva Millan, Ali Rezaie, Mark Pimentel

**Affiliations:** 1 Medically Associated Science and Technology (MAST) Program, Cedars-Sinai Medical Center, Los Angeles, CA, United States of America; 2 Division of Endocrinology, Diabetes, and Metabolism, Cedars-Sinai Medical Center, Los Angeles, CA, United States of America; 3 Division of Digestive and Liver Diseases, Cedars-Sinai Medical Center, Los Angeles, CA, United States of America; University of Lincoln, UNITED KINGDOM

## Abstract

Small intestinal bacterial overgrowth (SIBO) is highly prevalent and is associated with numerous gastrointestinal disorders, but the microbes involved remain poorly defined. Moreover, existing studies of microbiome alterations in SIBO have utilized stool samples, which are not representative of the entire gastrointestinal tract. Therefore, we aimed to determine and compare the duodenal microbiome composition in SIBO and non-SIBO subjects, using duodenal aspirates from subjects undergoing standard-of-care esophagogastroduodenoscopy without colon preparation. Using the recently-redefined cutoff for SIBO of >10^3^ colony forming units per milliliter (CFU/mL), 42 SIBO and 98 non-SIBO subjects were identified. Duodenal samples from SIBO subjects had 4x10^3^-fold higher counts than non-SIBO subjects when plated on MacConkey agar (P<0.0001), and 3.8-fold higher counts when plated on blood agar (P<0.0001). Twenty subjects had also undergone lactulose hydrogen breath tests (LHBTs), of whom 7/20 had SIBO. At the 90-minute timepoint, 4/7 SIBO subjects had positive LHBTs (rise in hydrogen (H_2_) ≥ 20 ppm above baseline), as compared to 2/13 non-SIBO subjects. 16S ribosomal RNA (rRNA) sequencing revealed that SIBO subjects had 4.31-fold higher relative abundance of Proteobacteria (FDR P<0.0001) and 1.64-fold lower Firmicutes (P<0.0003) than non-SIBO subjects. This increased relative abundance of Proteobacteria correlated with decreased α-diversity in SIBO subjects (Spearman R = 0.4866, P<0.0001) Specific increases in class Gammaproteobacteria correlated with the area-under-the-curve for H_2_ for 0–90 mins during LHBT (R = 0.630, P = 0.002). Increases in Gammaproteobacteria resulted primarily from higher relative abundances of the family Enterobacteriaceae (FDR P<0.0001), which correlated with the symptom of bloating (Spearman R = 0.185, 2-tailed P = 0.028). Increases in family Aeromonadaceae correlated with urgency with bowel movement (Spearman R = 0.186, 2-tailed P = 0.028). These results validate the >10^3^ CFU/mL cutoff for the definition of SIBO, and also reveal specific overgrowth of Proteobacteria in SIBO vs. non-SIBO subjects, coupled with an altered Proteobacterial profile that correlates with symptom severity. Future research may elucidate host-microbiome interactions underlying these symptoms in SIBO patients.

## Introduction

Small intestinal bacterial overgrowth (SIBO) is a condition defined by abnormal and excessive numbers of bacteria in the small bowel, usually associated with gastrointestinal (GI) symptoms such as bloating, excess of gas, abdominal discomfort, diarrhea, and weight loss [1, 2]. Interest in SIBO has been fueled by the ever-increasing awareness of the human microbiome and its potential relationships to human health and disease. SIBO is known to play important roles in a variety of conditions and disease states, including irritable bowel syndrome (IBS) [3, 4], fatty liver [5], inflammatory bowel disease [6], and many others [7], and associated symptoms include bloating, changes in bowel function and abdominal discomfort [1, 8, 9]. Although SIBO may represent the most common microbiome perturbation in medicine, it was recently noted that the ‘spectrum of SIBO’ remains to be truly defined [10], in part due to the lack of understanding of the normal bacterial populations of the small intestine, and how these may be altered in disease states [10].

Traditionally, SIBO has been defined by two primary methods; direct culture of small bowel aspirates [11], and indirect assessment using carbohydrate (e.g. lactulose, glucose) breath testing [12, 13]. In their current forms, both tests provide only a narrow understanding of the composition of the small intestinal microbiome or its impact on the host. In the case of culture, there are many limitations. First, there is a need for validated techniques for sterile aspiration of small bowel fluid without contamination from oral microbiota [14, 15]. Second, the ideal sampling location within the 20-foot length of the small bowel is subject to question [16]. Furthermore, even after this invasive procedure to procure samples, the previous definition of SIBO by culture, >10^5^ colony forming units per milliliter (CFU/per mL) of aspirate, was based on older studies in subjects with post-surgical anatomy with blind loop syndrome, rather than normal anatomy [11]. Following an evidence-based assessment, a recent consensus re-established the definition of SIBO as >10^3^ CFU/mL based on normal controls [17]. In the case of breath testing, the lack of a gold standard has left critics with concerns as to what a breath test means [2]. However, breath testing has been shown to have great value when comparing diseases such as IBS to healthy subjects [3] and there is growing interest in assessing methane as a marker of constipation [18], as well as more recently in determining the role of hydrogen sulfide [19]. Despite the limitations of these diagnostic approaches, there is no doubt that patients with common conditions respond to antibiotics, supporting a role for the microbiome in certain conditions and even leading to approval by the United States Food and Drug Administration (FDA) of an antibiotic for the treatment of IBS [20].

In tandem with this increased study of SIBO, there has been an exponential growth in the understanding of the human microbiome as important to health and disease [21–24]. This interest has been fueled by the emergence of more advanced and rapid sequencing technologies and methods of classifying the microbes that colonize the human body. While these advances have rapidly increased our understanding of the gut microbiome, almost the entire body of literature is based on stool or distal colonic assessments [21–24]. The small intestine, which has largely been omitted, is the longest segment of the gut and is responsible for nutrient absorption as well as control of numerous nutrient-driven endocrine and exocrine signaling pathways [25, 26], and maturation/modulation of the immune system [27, 28]. The REIMAGINE (Revealing the Entire Intestinal Microbiota and its Associations with the Genetic, Immunologic, and Neuroendocrine Ecosystem) study is the first large-scale initiative to map the small bowel microbiome, in order to examine the importance of these microbes in the context of disease [29]. In this study, we aimed to better define SIBO using 16S ribosomal RNA (rRNA) sequencing of the duodenum. The results more accurately and completely elucidate the microbial composition of the small bowel and how it is altered in SIBO.

## Materials and methods

The REIMAGINE study seeks to explore the roles of the small bowel microbiome in human health and disease. Specifically, this large-scale study involves collecting data from consecutive patients undergoing upper gastrointestinal endoscopy (esophagogastroduodenoscopy) (EGD) without colonoscopy for standard of care purposes, including GI symptoms, screening, anemia, GI bleeding, and to rule out celiac disease. All subjects complete a comprehensive health information questionnaire, after which small bowel aspirates are collected [29]. The study protocol was approved by the Cedars-Sinai Medical Center Institutional Review Board, and all subjects provide informed written consent prior to participation in the study.

### Study subjects

Male and female subjects aged 18–85 years undergoing standard of care EGD without colon preparation were prospectively recruited. Study staff identified potential participants, after which co-investigators or the principal investigator verified their eligibility. Subjects undergoing antibiotic therapy at the time of EGD were excluded from the analyses for the present study.

### Study procedures

#### Questionnaires

Prior to endoscopy, all subjects completed a study questionnaire which documented their demographics and medical and family history, including GI disease and bowel symptoms, use of medications, use of alcohol and recreational drugs, travel history, and dietary habits and changes. Medical information provided by participants was verified using medical records audits on all subjects. All patient data were de-identified prior to analysis. The severity of GI symptoms was indicated using a scale of 0 (absent symptom) to 100 (extremely severe), including abdominal pain, bloating, diarrhea, excess of gas, constipation, urgency with bowel movement and straining during bowel movement.

#### Breath testing

A subset of subjects had undergone standard of care lactulose hydrogen breath tests (LHBTs), and these were available for analysis. During this test, subjects were fasted for 12 hours, after which breath samples were collected at baseline and then every 15 minutes after the ingestion of 10g of lactulose, for up to 120 total minutes. At each timepoint, hydrogen (H_2_) and methane (CH_4_) were measured using a Quintron BreathTracker (Quintron Instrument Company, Milwaukee, WI, USA), using carbon dioxide (CO_2_) measurements to correct for alveolar gas concentration as per the manufacturer’s instructions. A positive breath test for SIBO was defined as a rise in H_2_ of ≥ 20 parts per million (ppm) above the baseline value by 90 minutes following substrate ingestion [17]. Additionally, a sensitivity analysis was conducted around the cut-off of H_2_ of ≥ 20 ppm above the baseline to validate the sensitivity and specificity of the breath test at different time-points after lactulose ingestion.

#### Small intestinal sample procurement

During the EGD procedure, luminal fluid samples were procured from the duodenum using a custom-designed sterile aspiration catheter (Hobbs Medical, Inc.). The endoscopist entered the second portion of the duodenum and inserted the aspiration catheter, and the inner catheter aspirated duodenal fluid through side holes to obtain a volume of 1–2 mL. The inner catheter was sterile and was pushed through a sterile bone wax cap only when in the duodenum. This was done to prevent any contamination from the scope as it traversed the proximal portions of the GI tract [29].

#### Aspirate processing and microbial culture

Prior to microbial culture, duodenal aspirates (DA) were processed with sterile 1x dithiothreitol (DTT) (1:1 ratio) and vortexed until the sample was completely liquified (~30 seconds), per our published protocol [29]. 100 μl of this mixture was serially diluted with 900 μl sterile 1x phosphate-buffered saline (PBS) and samples of both the 1:10 and the 1:100 dilutions were plated in duplicate on MacConkey agar (Becton Dickinson, Franklin Lakes, NJ, USA), and on blood agar (Becton Dickinson). MacConkey plates were incubated aerobically at 37°C for 16–18 hours, and blood agar plates were incubated anaerobically at 37°C for 16–18 hours in a rectangular anaerobic jar containing Mitsubishi™ AnaeroPack-Anaero (Thermo Scientific) packs. If no colonies were observed after 16–18 hours, plates were incubated for an additional 24 hours. Colonies were counted electronically using the Scan 500 (Interscience, Paris, France). As negative controls, 100μl aliquots of 1x DTT were cultured on MacConkey agar and on blood agar. SIBO was defined as the presence of >10^3^ colony-forming units (CFU) per mL of aspirate on MacConkey agar plates [17].

After microbial culture, the remainder of each DA was centrifuged at >17000 x g for 10 minutes. The supernatant was removed, and 500 μl of sterile All Protect reagent (Qiagen, Hilden, Germany) was added to each pellet to stabilize DNA, RNA and proteins. Pellets were stored at -80°C prior to DNA isolation and analysis of the duodenal microbiome.

#### DNA extraction and quantification from aspirates

DA samples were thawed on ice and an equal volume of 1x DTT was added, per our published protocol [29]. Samples were vortexed until the All Protect reagent was completely liquefied (~30 seconds). Samples were centrifuged at high speed and the supernatant was discarded. DNA extractions were performed using the MagAttract PowerSoil DNA KF Kit (Qiagen, cat. No. 27000-4-KF), again using aliquots of 1x DTT as negative controls.

The lysis step was performed by adding garnet beads (Qiagen, cat. No. 13123–50), 746 μl PowerBead Solution, 4 μl RNase A, and 60 μl SL Solution (Lysis buffer) to each tube. Tubes were sealed, vortexed horizontally for 15 minutes, and centrifuged for 6 minutes at 4500 x g. Subsequent steps were carried out according to the manufacturer’s instructions (Qiagen). DNAs were purified in 96 well plates using the KingFisher Duo (Thermo Fisher Scientific, Waltham, MA, USA) and quantified using Qubit ds DNA BR Assay kits (Invitrogen by Thermo Fisher Scientific) on a Qubit 4 Fluorometer (Invitrogen) [29].

#### Library preparation

16S library preparation was performed and the hypervariable V3 and V4 regions were amplified as described previously [29], using gene-specific primers (S-D-Bact-0341-b-S-17 and S-D-Bact-0785-a-A-21) described by Klindworth et al. [30]. Illumina sequencing adapters were added to each primer.

The full primer sequences were as follows:

16S Amplicon PCR Forward Primer: 5'TCGTCGGCAGCGTCAGATGTGTATAAGAGACAGCCTACGGGNGGCWGCAG

16S Amplicon PCR Reverse Primer: 5'GTCTCGTGGGCTCGGAGATGTGTATAAGAGACAGGACTACHVGGGTATCTAATCC

16S library preparation was performed by adding 5 μl of DNA to a Master Mix containing 0.5 μl 10 μM 16S Amplicon PCR Forward primer, 0.5 μl 10 μM 16S Amplicon PCR Reverse primer, 12.5 μl 2x KAPA HiFi HotStart ReadyMix and 6.5 μl of molecular grade PCR H_2_O [29]. The first round of PCR consisted of 27 cycles, and was followed by an optimized Clean-Up step utilizing Agencourt AMPure XP beads following a protocol described by Quail et al. [31].

The second round of PCR was performed using 5 μl of the final Amplicon PCR products with the Nextera XT Index kit and 2x KAPA HiFi HotStart ReadyMix, in accordance with Illumina’s published protocol (https://support.illumina.com/documents/documentation/chemistry_documentation/16s/16s-metagenomic-library-prep-guide-15044223-b.pdf). Another modified Clean-Up step was performed, after which the final products were quantitated using Qubit 1X dsDNA HS Assay kits on a Qubit 4 Fluorometer and then analyzed with Agilent DNA 1000 chips (Agilent Technologies, Santa Clara, CA, USA) on the Agilent 2100 Bioanalyzer System.

#### 16S rRNA gene sequencing and analysis

V3 and V4 libraries were sequenced with a MiSeq Reagent Kit v3 (600-cycles) on a MiSeq System (Illumina, San Diego, CA, USA) [29]. 2x301 cycles of paired-end sequencing were performed, with 5% Phix (Illumina) being added to each library pool.

Operational Taxonomic Unit (OTU) clustering and taxonomic analyses were performed using CLC Genomics Workbench v. 10.1.1 and CLC Microbial Genomics Module v. 2.5 (Qiagen). Sequences were trimmed, merged and clustered into OTUs at 97% sequence similarity with the Amplicon-Based OTU clustering tool. The most abundant sequences were selected as representative of each cluster, and taxonomic levels were assigned using CLC Microbial Genomics default values by comparing against the 2013 Greengenes Database release. Low depth samples (< 9,000 sequences per sample) were removed, and alpha diversity indexes were calculated. The weighted Unifrac metric was employed for the calculation of inter-sample diversity (beta diversity).

### Statistics

Predictions for significant differentially abundant OTUs between non-SIBO and SIBO subjects were performed following recommendations from McMurdie and Holmes [32], and from Weiss et al. [33], used when the average library size for each group is approximately equal and/or the fold difference between groups is not high (>2-3x on average).

Multiple comparisons and statistical analyses were carried out with CLC Genomics Workbench v. 10.1.1 and CLC Microbial Genomics Module v. 2.5 (Qiagen). A Negative Binomial GLM model was used to obtain maximum likelihood estimates for the fold change (FC) of an OTU between two conditions, and the Wald test was used for determination of significance. False Discovery Rate (FDR) was performed to correct the p-values. Fold changes are calculated from the generalized linear model (GLM), which corrects for differences in library size between the samples and the effects of confounding factors.

Two-tailed Spearman R correlations, Mann-Whitney tests and graph construction were carried out with rarefied OTU tables using GraphPad Prism 7.02 (GraphPad Software, La Jolla, CA, USA) [34]. For the purposes of statistical analysis only, no growth on blood agar or MacConkey agar (i.e. CFU/mL = 0) was designated as 1 CFU/mL. Breath testing and sequencing results were compared by correlating the area-under-the-curve (AUC) for H_2_ at different timepoints with relative abundances of bacterial populations previously associated with SIBO. Sensitivity analyses were performed in order to determine whether 90 minutes was the ideal timepoint for the prediction of SIBO.

#### PICRUSt analysis for predicted metabolic functions

Predicted metabolic functions in the identified microbial communities were determined using the Phylogenetic Investigation of Communities by Reconstruction of Unobserved States package (PICRUSt v1.1.3) [35]. The analysis was performed using OTUs closed-referenced picked against the newest version of the Greengenes database at 97% identity. Metabolic functions were predicted using the Kyoto Encyclopedia of Genes and Genome (KEGG) Orthology (KO) Database7 [36, 37]. PICRUSt accuracy was estimated using weighted Nearest Sequenced Taxon Index (weighted NSTI) values. Low NSTI values imply the predicted KEGG functional groups are highly accurate [35]. Statistical comparisons of predicted metabolic functions in DA groups were performed using the Mann-Whitney test in IBM^®^ SPSS^®^ version 24.

Heatmaps were constructed and hierarchical clustering of predicted metabolic functions was performed considering Spearman rank correlation and complete linkage methods using Morpheus software (https://software.broadinstitute.org/morpheus/).

### Data and materials availability

Data for this project are available at the National Center for Biotechnology Information (NCBI) under BioProject ID PRJNA525828.

## Results

### Microbial culture and definition of SIBO

Duodenal aspirates (DA) from a total of 140 consecutive subjects were collected and analyzed (Table 1). A total of 98 subjects were defined as non-SIBO, based on bacterial counts ≤10^3^ CFU/mL for DA when plated on MacConkey agar. The remaining 42 subjects had bacterial counts >10^3^ CFU/mL on MacConkey agar and were defined as having SIBO [17]. This cutoff distinguished two distinct groups of subjects, as DA from SIBO subjects had 4x10^3^-fold higher bacterial counts on MacConkey agar than DA from non-SIBO subjects (P<0.0001). DA from SIBO subjects also had markedly higher numbers of anaerobes when plated on blood agar compared to DA from non-SIBO subjects (FC = 3.08, P<0.0001). The prevalence of prior use of antibiotics and current treatment with proton pump inhibitors (PPI) were not confounders, as both exhibited similar prevalence in subjects with and without SIBO (P = 0.134, OR: 1.8 95% Cl 0.889 to 3.636 for prior antibiotics; P>0.999, OR: 1.04 95% Cl 0.455 to 2.275 for PPI use, respectively) (Table 1).

**Table 1:** Demographics for non-SIBO and SIBO subjects.

	Non-SIBO (N = 98)	SIBO (N = 42)	P-value
Gender (Male/Female)	40/58	17/25	P = 0.6129*
Age (Mean ± SD)	57.93 ± 15.84	63.09 ±14.14	P = 0.0644^#^
Prior use of antibiotics (%)	47.7	33.7	P = 0.1335^a^
Current use of PPI (%)	27.3	26.5	P>0.999^a^

* Chi-Square test,

^#^ t-test,

^a^ Fisher’s exact test.

### Utility of breath tests for detection of SIBO

Twenty (20) of the 140 subjects had also undergone standard of care breath testing. Of these, seven subjects (7/20) had SIBO based on the threshold of >10^3^ CFU/mL on MacConkey agar. Four of these seven (4/7) SIBO subjects also had a positive H_2_ breath test at 90 minutes (defined as a ≥20ppm rise in H_2_ levels from baseline), indicating a 57.14% sensitivity for the detection of SIBO. Thirteen subjects (13/20) did not have SIBO based on the >10^3^ CFU/mL threshold, and of these, two (2/13) had a positive H_2_ breath test at 90 minutes, indicating a 84.61% specificity for the detection of SIBO. A sensitivity analysis around H_2_ levels was performed for the 60, 75, 90, 105, and 120-minute time points, again using the threshold of a ≥20ppm rise in H_2_ levels from baseline to define positivity. A positive breath test (rise in H_2_ of ≥20ppm) at 75 minutes had the same sensitivity and specificity for the detection of SIBO as a positive breath test at 90 minutes, with lesser sensitivities and/or specificities for the other time points (see Table 2).

**Table 2:** Relationships between H_2_ levels, area under the curve (AUC) during breath testing, detection of SIBO, relative abundances of microbial taxa, and other parameters.

Timepoint (minutes)	SIBO detection		H_2_ AUC	Gammaproteobacteria abundance		Firmicutes abundance		Excess of gas		Energy metabolism (H_2_ production)	
	Sensitivity (%)	Specificity (%)		Spearman r	P-value	Spearman r	P-value	Spearman r	P-value	Spearman r	P-value
60	14.28	92.3	16.72	0.488	**0.021**	-0.318	0.149	0.355	0.105	0.272	0.221
75	57.14	84.61	24.06	0.516	**0.014**	-0.371	0.089	0.39	0.073	0.313	0.156
90	57.14	84.61	35.16	0.63	**0.002**	-0.508	**0.016**	0.408	0.059	0.457	**0.033**
105	57.14	69.23	49.06	0.689	**3.88E-04**	-0.583	**0.004**	0.444	**0.039**	0.527	**0.012**
120	57.14	61.53	52.97	0.781	**1.80E-05**	-0.651	**0.001**	0.368	0.092	0.602	**0.003**

Significant changes (P <0.05) are indicated in bold.

Darker green color represents greater sensitivity and specificity. Darker red color represents higher correlation.

### Decreased duodenal microbiome diversity in SIBO

**Fig 1:**
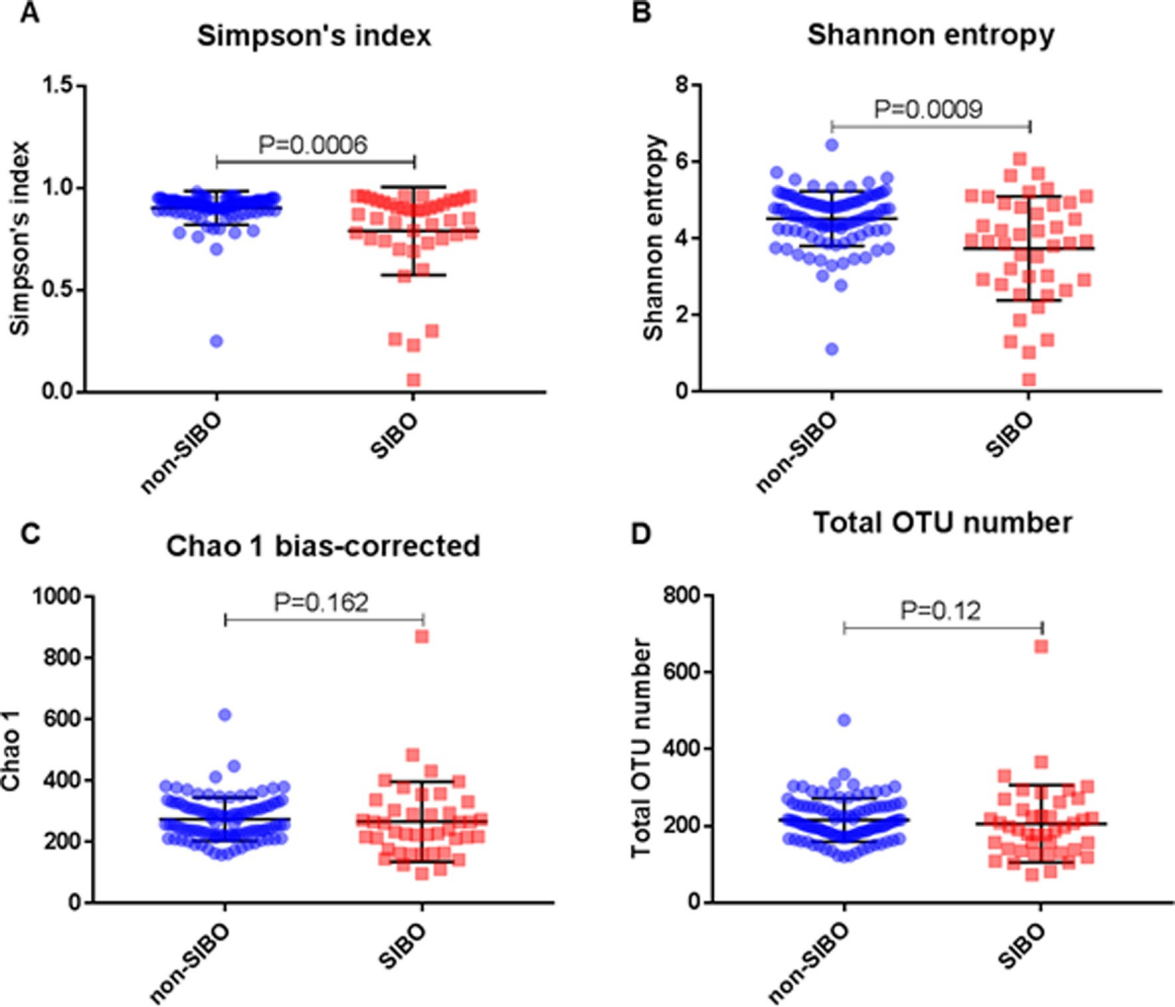
Alpha diversity of SIBO (N = 42) and non-SIBO (N = 198) subjects, as identified by microbial counts on MacConkey agar. A—Simpson’s index diversity; B—Shannon entropy diversity; C–Chao 1 diversity; D–Total number diversity.

All 140 DA underwent 16S rRNA gene sequencing (N = 42 SIBO; N = 98 non-SIBO). The difference in average library sizes between the SIBO and non-SIBO groups was less than 1.2-fold (S1 Table). The duodenal microbiome in SIBO subjects exhibited markedly decreased microbial α-diversity when compared to non-SIBO subjects, with significant differences in three of the five indices analyzed, including Simpson’s index (P = 0.0006) and Shannon entropy (P = 0.0009) (Fig 1). Differences in Chao 1 and Total OTU number did not reach significance (P = 0.162 and P = 0.12 respectively) (Fig 1).

### Duodenal microbiome differences in SIBO

**Fig 2:**
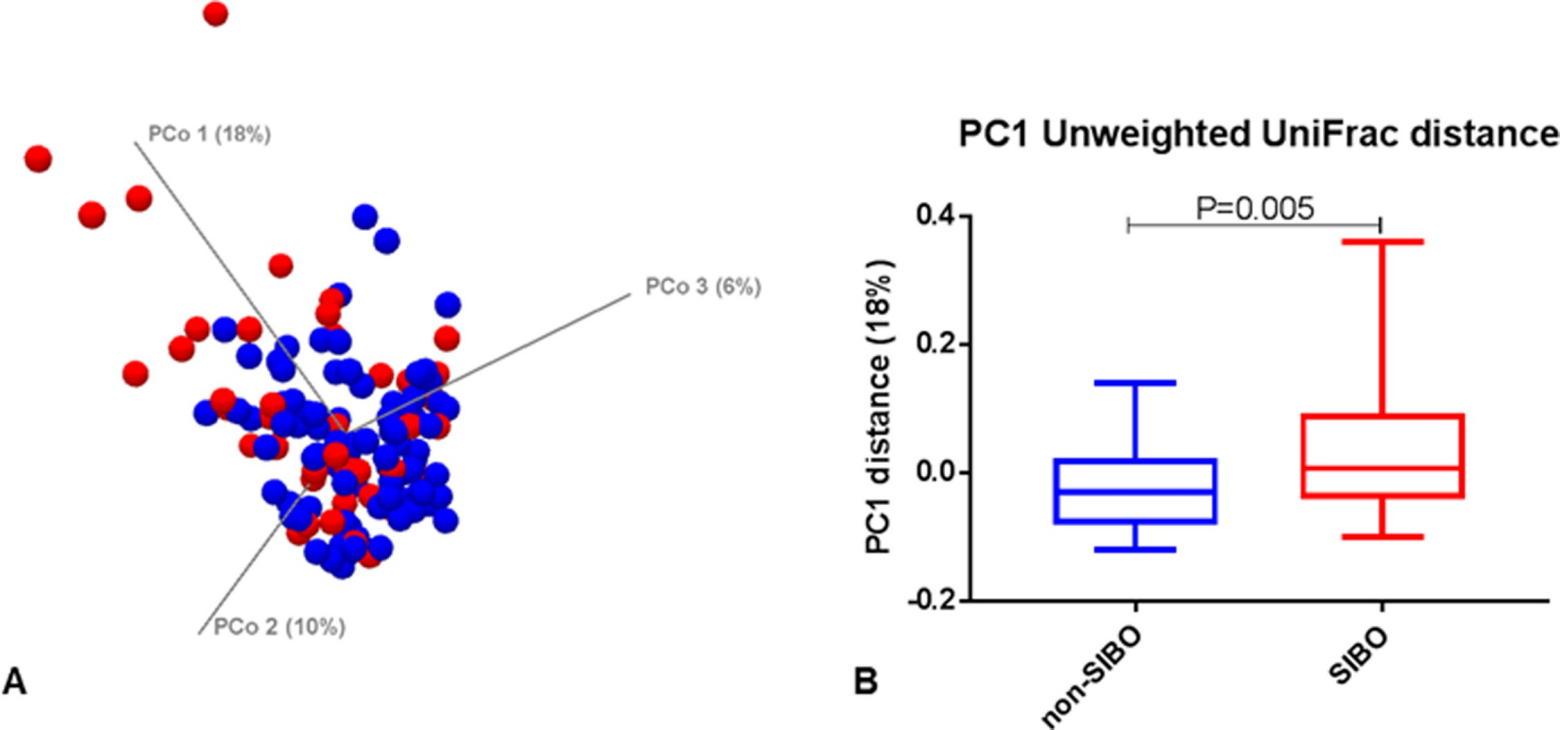
A- Principal Coordinates Analysis (PCoA) of both binary and abundance Unweighted UniFrac distances between SIBO (red) and non-SIBO (blue) subjects. B–PC1 distance plot.

Principal Coordinates Analysis (PCoA) of both binary and abundance Unweighted UniFrac distances between SIBO and non-SIBO subjects were different (PCo1 P = 0.005) (Fig 2). Many subjects with SIBO (N = 26) clustered in both PCoA second and third principal components (PCo2 and PCo3) (Fig 2).

#### Phylum level

**Fig 3:**
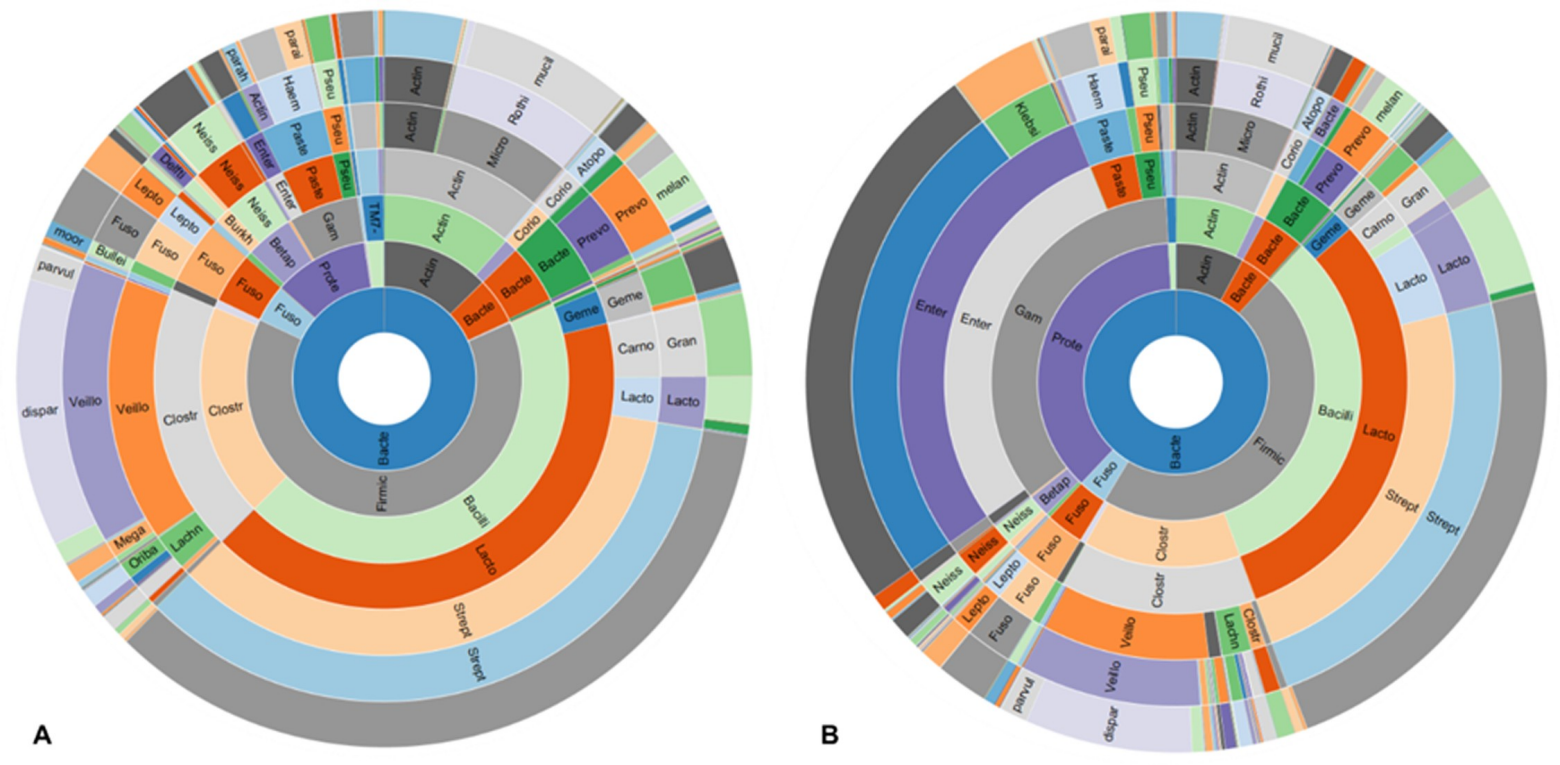
Sunburst representation of the duodenal microbiome in (A) non-SIBO (N = 98) and (B) SIBO (N = 42) subjects.

**Fig 4:**
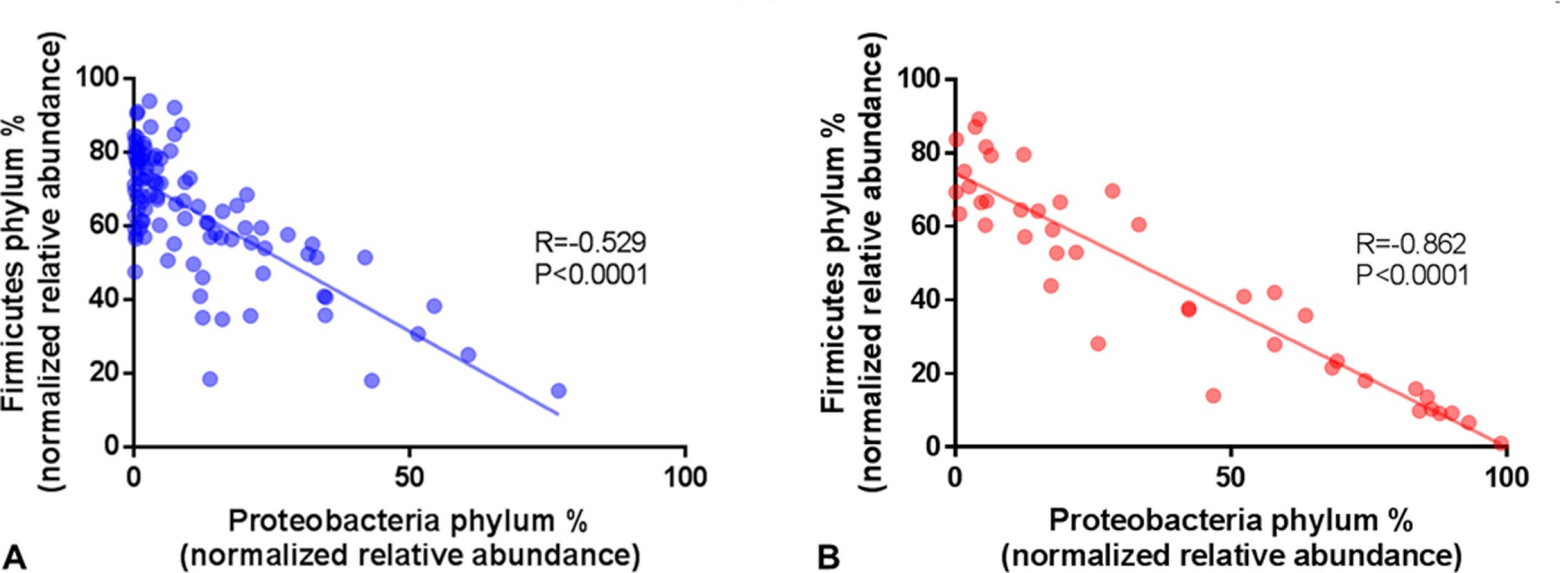
Correlation of the phyla Proteobacteria and Firmicutes in DA from (A) non-SIBO (N = 98) and (B) SIBO (N = 42) subjects.

**Fig 5:**
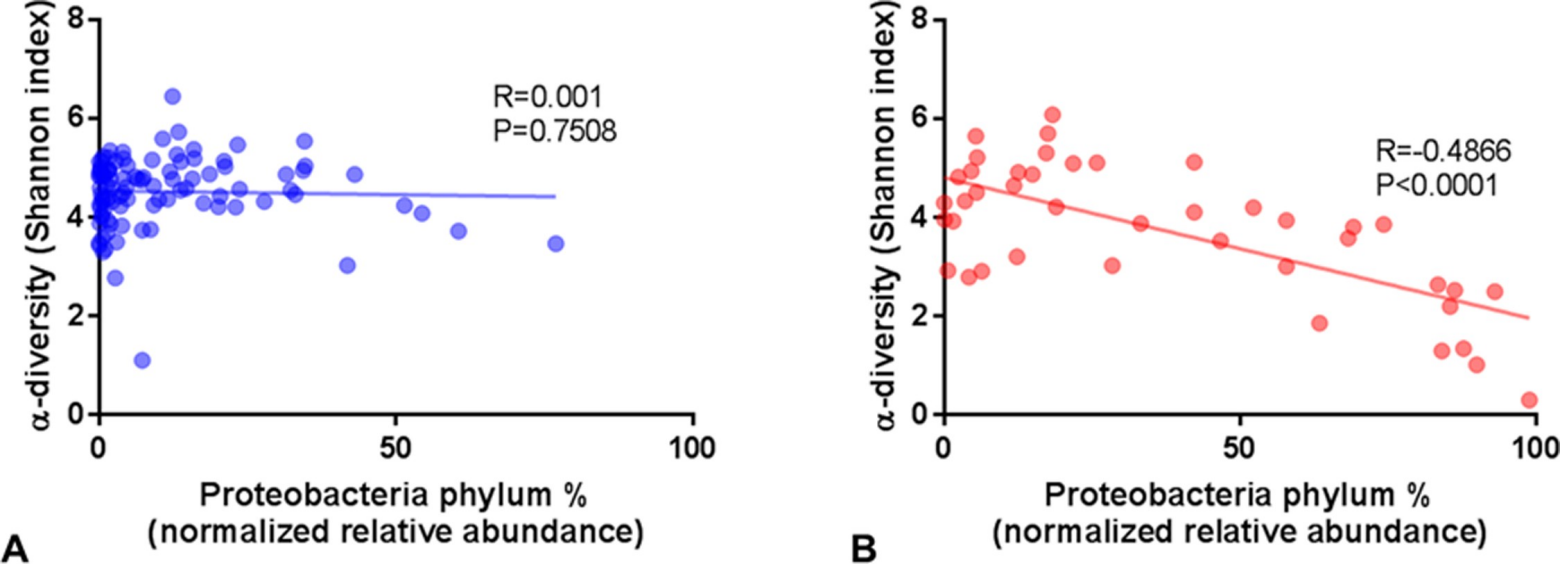
Correlation between the phylum Proteobacteria and α-diversity in DA from (A) non-SIBO (N = 98) and (B) SIBO (N = 42) subjects.

The three dominant phyla identified in DA from non-SIBO subjects were Firmicutes (64%), Actinobacteria (13%) and Proteobacteria (11%), followed by smaller proportions of Fusobacteria, Bacteroidetes and TM7 (Fig 3 and Table 3). The dominant phylum in DA from SIBO subjects was Proteobacteria (37%), which was 3.19-fold higher than in non-SIBO subjects (FDR 2.21E-14) (Fig 3 and Table 3). This increased relative abundance of Proteobacteria in SIBO subjects was associated with a decreased relative abundance of Firmicutes (FC = -1.36, P = 0.0007), and to a lesser extent of other phyla (Table 3). In fact, there was a significant strong inverse correlation between the phyla Proteobacteria and Firmicutes in subjects with SIBO (Spearman R = -0.8622, P<0.0001) (Fig 4A). This was also seen in non-SIBO subjects, but only to a moderate extent based on R-value (Spearman R = -0.5297, P<0.0001) (Fig 4B). The Firmicutes/Proteobacteria ratio in SIBO subjects (Med = 2.19, 25^th^ Percentile = 0.302, 75^th^ Percentile = 11.72) was 7.33-fold lower than in non-SIBO subjects (Med = 16.05, 25^th^ Percentile = 3.39, 75^th^ Percentile = 62.89) (P<0.0001). In fact, using a Proteobacteria/Firmicutes ratio of greater than 0.39 as a cutoff for the determination of SIBO based on sequencing resulted in a sensitivity of 54.76% (95% confidence interval (CI): 38.67% to 70.15%) and a specificity of 81.63% (95% CI: 72.53% to 88.74%) compared to determination based on microbial culture (odds ratio (OR):5.38, Fisher’s exact test P<0.0001). Interestingly, relative abundance of Proteobacteria was associated with the decreased α-diversity observed in subjects with SIBO (Spearman R = 0.4866, P<0.0001) (Fig 5).

**Table 3:** Relative abundances of the top six phylae in the duodenal microbiomes of non-SIBO and SIBO subjects.

	Non-SIBO (N = 98)	SIBO (N = 42)	Non-SIBO vs. SIBO		
Phylum	Relative abundance (%)	Relative abundance (%)	Fold Change	P-value*	FDR P-value**
Firmicutes	64	47	-1.36	**0.0007**	0.4
Actinobacteria	13	8	-1.59	**0.0004**	0.36
Proteobacteria	11	37	3.19	**<0.0001**	**2.21E-14**
Fusobacteria	4	3	-1.34	**0.0062**	0.9
Bacteroidetes	6	4	-1.43	0.05	0.9
TM7	2	1	-2.3	**0.0004**	0.14

Significant changes (P-value<0.05 and FDR P-value<0.05) are indicated in bold.

*—Mann-Whitney test;

**—Wald-test.

#### Class level

At the class level, the relative abundance of the class Gammaproteobacteria, which is one of the most important classes in the phylum Proteobacteria and includes several clinically relevant pathogens, was increased in DA from SIBO subjects compared to non-SIBO subjects (FC = 4.73, FDR P<0.0001). This increase was accompanied by increases in class Deltaproteobacteria (FC = 3.07, FDR P = 2.07E-7) (S2 Table). The relative abundance of the class Gammaproteobacteria also exhibited a strong inverse correlation with levels of the phylum Firmicutes (Spearman R = -0.917, P<0.0001) in subjects with SIBO, suggesting the Proteobacteria effect was driven by the class Gammaproteobacteria.

#### Family and genus level

At the family level, the increased abundance of the class Gammaproteobacteria in DA from SIBO subjects was driven by higher relative abundances of Enterobacteriaceae (FC = 7.35, FDR P<0.0001), which represented 89% of the total relative abundance of Gammaproteobacteria in SIBO subjects, as well as increased relative abundances of the families Aeromonadaceae (FC = 4.87, FDR P = 2.0E-10) and Moraxellaceae (FC = 8.31, FDR P<0.0001). Moreover, the relative abundances of these families exhibited associations with self-reported gastrointestinal symptoms in these subjects. The relative abundance of the family Enterobacteriaceae exhibited a positive correlation with bloating (Spearman R = 0.185, 2-tailed P = 0.028) and that of the family Aeromonadaceae correlated with urgency with bowel movement (Spearman R = 0.186, 2-tailed P = 0.028). Consistent with this, subjects with SIBO (as determined by microbial culture) also exhibited greater urgency with bowel movement than non-SIBO subjects (P = 0.022).

The relative abundances of several genera from these families were also increased in subjects with SIBO when compared to non-SIBO subjects, including *Klebsiella* and *Escherichia/Shigella* from family Enterobacteriaceae (FC = 7.8, FDR P<0.0001; FC = 7.18, FDR P<0.0001 respectively), an unknown genus from family Aeromonadaceae (FC = 3.87, FDR P = 6.57E-7), and *Acinetobacter* and an unknown genus from family Moraxellaceae (FC = 2.08, FDR P = 2.09E-3; FC = 9.33, FDR P<0.0001 respectively).

### Changes in microbial metabolic functions in SIBO

The predicted metabolic functions of the duodenal microbial communities from both groups were analyzed based on the 16S rRNA sequencing profiles. The NSTI value was determined in each group (SIBO NSTI = 0.04; non-SIBO NSTI = 0.055) and the mean value calculated to be 0.05 ± 0.03 SD for all subjects, indicating high accuracy of the metabolic function predictions. Hierarchical clustering of all subjects showed several subjects with SIBO clustering together based on predicted Kyoto Encyclopedia of Genes and Genome (KEGG) level 2 metabolic functions (S1 Fig). SIBO subjects exhibited a different microbe-associated KEGG level 2 metabolic profile when compared to non-SIBO subjects, with high number of sequences assigned to metabolism, including energy metabolism (P<0.0001), carbohydrate metabolism (P<0.0001), glycan biosynthesis and metabolism (P<0.0001), lipid metabolism (P<0.0001), and many other functions (see S3 Table for full description). The pattern of the sequences assigned to predicted metabolic functions appeared to be associated with the relative abundances of the phyla Firmicutes and Proteobacteria, and with α-diversity (S2 Fig). Low relative abundance of Firmicutes, high relative abundance of Proteobacteria and low α-diversity were associated with a variety of predicted metabolic functions, resulting in a distinct pattern observed on the hierarchical clustering analysis heatmap (S2 Fig).

Lastly, formate metabolism was analyzed using the presence of the formate dehydrogenase major unit gene (FdhF, K00123). The abundance of OTUs which contribute to formate metabolism was increased in the duodenal microbiome of SIBO vs. non-SIBO subjects (FC = 10.09, P = 0.0017). 59% of the OTUs that contributed to the presence of FdhF were from the phylum Proteobacteria, principally represented by members of the class Gammaproteobacteria (64%).

### Relationship between breath testing and SIBO in breath test subgroup

When analyzed relative to the area-under-the-curve (AUC) for H_2_ at different timepoints during the LHBT, the relative abundance of the class Gammaproteobacteria exhibited a positive correlation with the AUC for H_2_ at 90 minutes after lactulose ingestion (H_2_ AUC = 35.16, Spearman R = 0.630, 2-tailed P = 0.002). In contrast, the relative abundance of the phylum Firmicutes exhibited a negative correlation with the AUC for H_2_ at 90 minutes (Spearman R = -0.508, 2-tailed P = 0.016). AUCs for H_2_ at other timepoints correlated to a lesser extent with the relative abundance of the class Gammaproteobacteria, and also exhibited negative correlations with the relative abundances of the phylum Firmicutes at 105 minutes and 120 minutes (see Table 2).

Based on PICRUSt analysis of microbial metabolic functions, the AUC for H_2_ at 90 minutes after lactulose ingestion exhibited a positive correlation with energy metabolism (Spearman R = 0.457, p = 0.033), which includes metabolic functions associated with formate degradation and the formation of H_2_. The AUCs for H_2_ at 105 and 120 minutes also correlated with this metabolic function (105 min Spearman R = 0.527, P = 0.012; 120 min Spearman R = 0.602, P = 0.003) (Table 2). In contrast, the AUCs at 60 and 75 minutes did not exhibit any correlation with this metabolic function (Table 2).

Comparing the microbiome findings and breath testing, the definition of the optimal time point in the H_2_ breath test for the detection of SIBO was determined based on the variables shown in Table 2. The 75- and 90-minute time points exhibited higher sensitivity (57.14%) for SIBO detection when compared to 60 minutes, and higher specificity (84.61%) for SIBO detection when compared to 105 and 120 minutes. The 90-minute time point exhibited a higher correlation with the abundances of Gammaproteobacteria and Firmicutes compared to the 75-minute time point. In addition, the levels of H_2_ at 90 minutes correlated with energy metabolism and exhibited a positive correlation with excess of gas that approached significance (P = 0.059). Based on these data, the 90-minute time point was defined as the optimal time point in the H_2_ breath test for the detection of SIBO in this study.

## Discussion

The data presented here are part of the REIMAGINE study, a novel study which uses carefully validated techniques for the acquisition and processing of the unique low biomass material found in small bowel aspirates [38]. In the present study, these techniques were used to explore the microbial populations of the small intestine and identify how these are altered in SIBO. The findings include a number of observations that also support the culture threshold of >10^3^ CFU/mL for the definition of SIBO. This is also the first description of an increase in the relative abundance of the phylum Proteobacteria in SIBO, and describes a unique Proteobacterial profile in SIBO. Furthermore, this increase correlates with a decrease in the relative abundance of Firmicutes, which are a normal (and perhaps beneficial) component of the small intestinal gut microbiome [39]. Research on SIBO to date has focused on a broad overview of the condition based on breath testing, and to a lesser extent on aspiration and culture of small bowel samples. Most of the published literature has explored associations between SIBO and clinical conditions, with some successes e.g. in the examination of IBS with diarrhea [40, 41]. While culture has been routinely described as the gold standard for SIBO, there has been little validation of this technique, coupled with widespread use of poorly validated definitions for a positive culture based on older data [11]. Systematic reviews [11] and a recent consensus statement [17] have resolved these to some extent through evidence-based reviews of the literature. In the latter, experts used the approach of defining abnormal by first defining “normal”. Using this approach, SIBO was defined as >10^3^ CFU/mL. However, this new definition has not been examined by more sophisticated approaches until now.

In this component of the REIMAGINE study, the goal was to assess SIBO as an entity and to determine if it could be defined using microbiome analytic techniques. However, many aspects of acquiring and processing duodenal aspirates needed optimization. From the literature, techniques vary widely and there is a high risk of sample contamination. First, the REIMAGINE study developed and validated a protected catheter to prevent oral contamination when sampling the small bowel. Second, as small intestinal fluid is highly viscous and present challenges for conventional DNA isolation techniques, the REIMAGINE study developed and validated techniques for improved sample processing and DNA isolation tailored to the small bowel [38]. These improved approaches were used for this study on SIBO.

An interesting initial finding in this study are the culture results. While growth on MacConkey agar was used for the definition of SIBO, it was clear that SIBO was a distinct entity in two ways. First, when SIBO was present, there was a very large fold increase in the overall numbers of bacteria seen on MacConkey plates compared to non-SIBO samples–there was no gradual gradient, but rather a 4x10^3^-fold increase in bacterial numbers associated with the threshold of >10^3^ CFU/mL. Second, the presence of SIBO based on MacConkey agar was also predictive of a higher count on blood agar. Broadly speaking, growth on MacConkey agar represents Gram-negative aerobic bacilli, including those from the phylum Proteobacteria, whereas blood agar can support a broad range of anaerobic organisms [42]. Again, these data indicate that the duodenal microbial profile in SIBO is dramatically different from the profile in non-SIBO subjects.

16S rRNA sequencing provides even more significant results using >10^3^ CFU/mL as the cutoff for the definition of SIBO. As expected from the high levels of Gram-negative bacilli seen on MacConkey plates, the composition of the duodenal microbiome as identified by sequencing revealed a significantly increased relative abundance of Proteobacteria in subjects with SIBO. A previous study demonstrated increased levels of Proteobacteria species including *Escherichia sp*, *Klebsiella sp*, and *Aeromonas sp* in IBS subjects compared to healthy controls, which is consistent with our findings as SIBO is often associated with IBS [43]. However, our findings have shown that this overgrowth of Proteobacteria is associated with a markedly reduced relative abundance of Firmicutes. Since Firmicutes are typical colonizers of the gut in healthy subjects [39], this may have health consequences that require further exploration. Previous studies indicated that an overgrowth of Proteobacteria in the gut (particularly Enterobacteriaceae) was associated with a concomitant depletion of Firmicutes in gut inflammatory diseases [44, 45].

Detailed examination of the Proteobacteria profiles in SIBO and non-SIBO subjects show that not only is there a greater abundance of Proteobacteria in SIBO, but the taxonomic composition of this phylum is also significantly altered. This is characterized by an increased relative abundance of class Gammaproteobacteria and a reduction in class Alphaproteobacteria. *Klebsiella* species and other representative of the family Enterobacteriaceae (class Gammaproteobacteria) were increased in subjects with SIBO, as were species from the genus *Aeromonas* (family Aeromonadaceae). This is interesting as many *Aeromonas* species are known to be mild pathogens [46]. These findings are consistent with previous studies indicating that the bacterial overgrowth in SIBO is due in part to organisms typically found in the colon [47], such as Enterobacteriaceae.

Proteobacteria is a major phylum of Gram-negative bacteria, and includes several known human pathogens [48]. Independent evidence suggests alterations in Proteobacteria as a possible microbial marker for dysbiosis of the gut microbiota and a common factor in human diseases [49, 50]. Proteobacteria-associated dysbiosis is the result of an overgrowth of representatives of this phylum, especially Enterobacteriaceae, reducing microbial diversity in the gut. In this study, subjects with SIBO exhibit lower microbial diversity associated with an increased abundance of Proteobacteria in the small intestine, revealing once again that overgrowth of this phylum impairs gut microbiome health. Proteobacteria overgrowth and low gut microbial diversity are also observed in undernourished children and inflammatory bowel disease (IBD) [49, 50].

The PICRUSt analysis provides further resolution on the importance of defining SIBO with more granularity. It is clear from this analysis that SIBO is associated with several downstream metabolic functions that could be connected to the clinical findings in these patients. In this study, H_2_ levels detected on breath testing correlated with the self-reported symptom excess of gas, and also correlated with microbial energy metabolism in the small bowel. Pathways involved in formate metabolism were also enriched in subjects with SIBO. Formate is the signature compound in the anaerobic metabolism of enterobacteria and its metabolism is associated with H_2_ production [51]. These associations reveal an important link between metabolites produced by Proteobacteria species and symptoms observed in subjects with SIBO.

In addition, and again supporting the link (possibly a causal relationship) between overgrowth of Proteobacteria and gastrointestinal symptoms observed in subjects with SIBO, clinically important families from this phylum were associated with symptoms commonly observed in subjects with SIBO, such as bloating (family Enterobacteriaceae) and urgency with bowel movement (family Aeromonadaceae).

While this is a comprehensive 16S sequencing validation of SIBO, there are some limitations. Principally, the comparison to breath testing is limited by the small number of patients who had breath testing. Despite this low number, there was a direct correlation between SIBO factors and hydrogen on breath test as well as metabolic analyses, supporting that the microbiome of SIBO has upregulated pathways that lead to hydrogen production.

In summary, this study describes an in-depth analysis of the small bowel microbiome in SIBO using validated and optimized small bowel microbiome assessment techniques. This study demonstrates that the cutoff of >10^3^ CFU/mL for the definition of SIBO is important in identifying a unique group of subjects defined not only by a higher relative abundance of Proteobacteria but also by a unique Proteobacterial profile. These results are enhanced by the identification of downstream pathways that are altered in the small bowel microbiome in SIBO. Moreover, the microbiome-associated findings correlate with clinical symptoms in subjects with SIBO. Exploration of these findings in future research may reveal novel host-microbiome interactions which account for the symptoms in these patients.

## Supporting information

S1 Fig Hierarchical clustering of KEGG level 2 microbial metabolic functions in non-SIBO (n = 98) and SIBO (n = 42) subjects. (PDF)

S2 Fig Hierarchical clustering of KEGG level 2 microbial metabolic functions, Proteobacteria phylum, Firmicutes phylum and Shannon diversity index in SIBO (n = 42) subjects. (TIF)

S1 Table 16S library sizes for DA from non-SIBO and SIBO subjects. (DOCX)

S2 Table Fold change in the relative abundance of Proteobacteria classes in DA from non-SIBO and SIBO subjects. (DOCX)

S3 Table Picrust analysis KEGG 2 pathway of subjects with SIBO compared to non-SIBO subjects. (DOCX)
