# Supplementary figures and images for: The duodenal microbiome is altered in small intestinal bacterial overgrowth
Source: PLoS One. 2020 Jul 9;15(7):e0234906. doi: 10.1371/journal.pone.0234906 (PMC7347122; doi:10.1371/journal.pone.0234906)

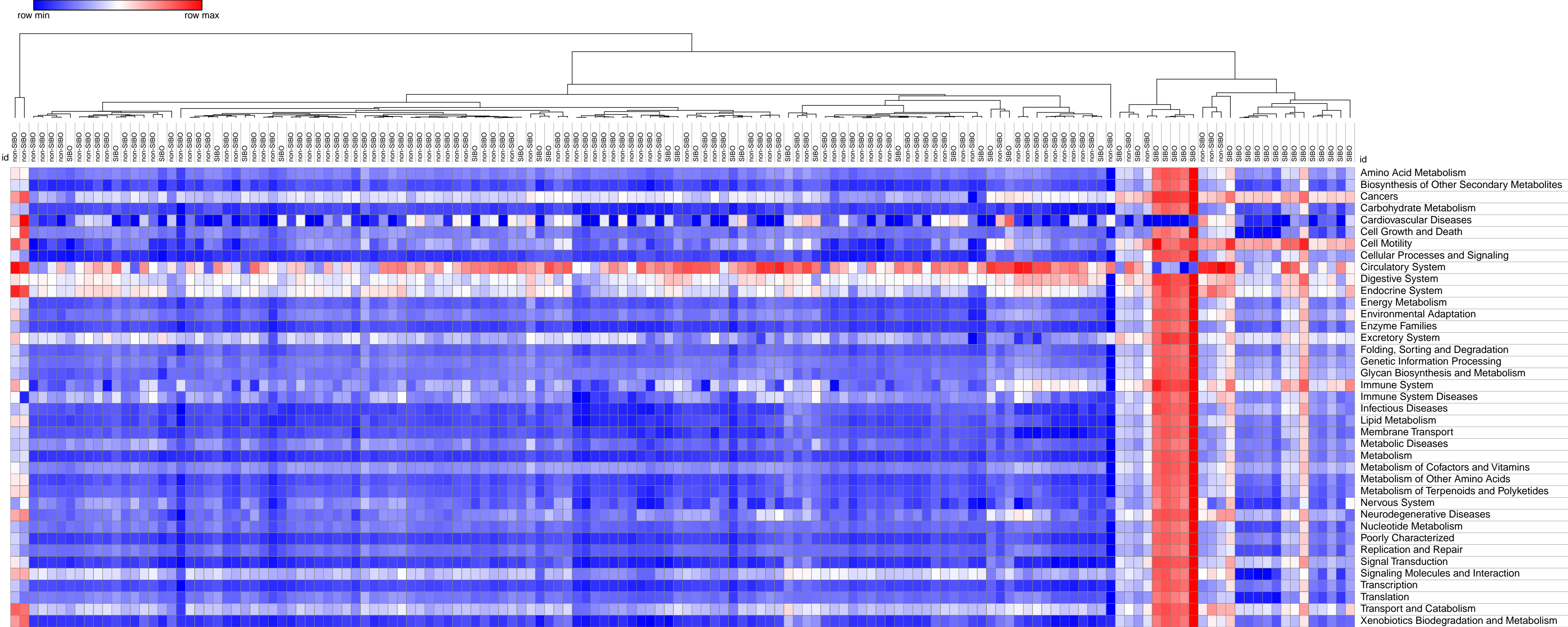

Supplement: S1 Fig — (PDF) [file pone.0234906.s001.pdf]

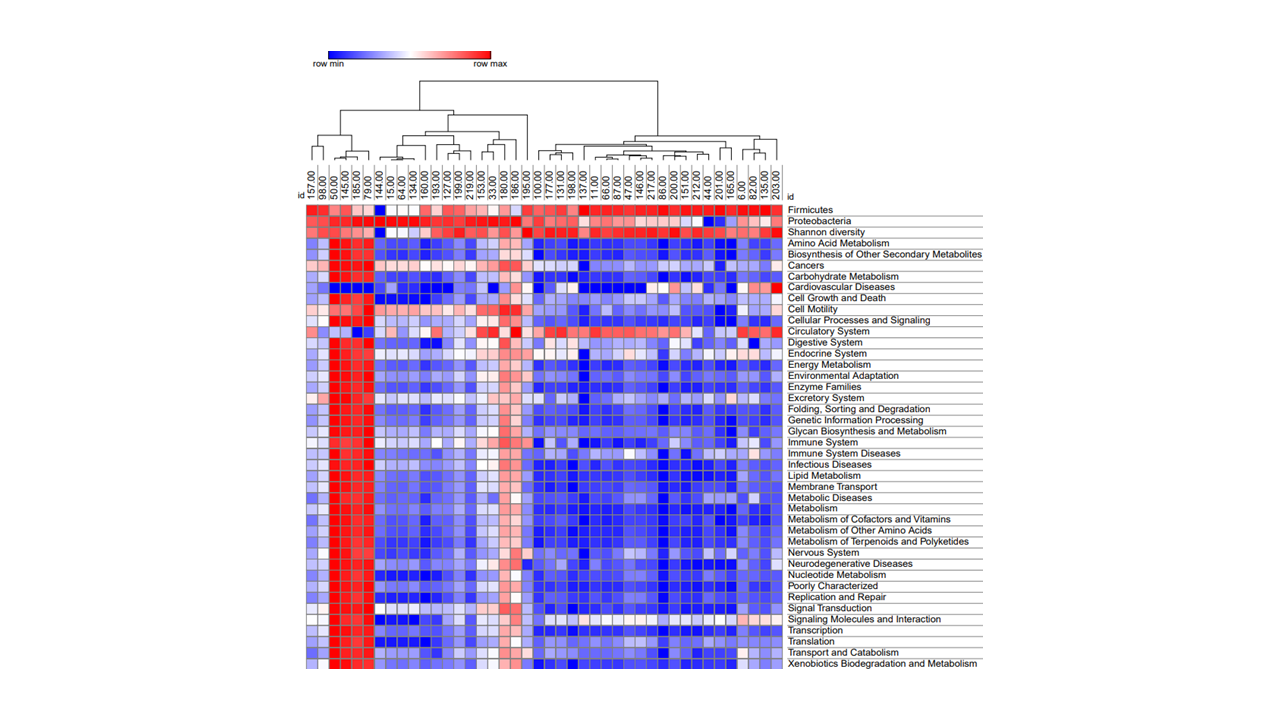

Supplement: S2 Fig — (TIF) [file pone.0234906.s002.tif]
