# Supplementary material for: The duodenal microbiome is altered in small intestinal bacterial overgrowth
Source: PLoS One. 2020 Jul 9;15(7):e0234906. doi: 10.1371/journal.pone.0234906 (PMC7347122; doi:10.1371/journal.pone.0234906)
Supplement: S1 Table — (DOCX) [file pone.0234906.s003.docx]

**S1 Table.** 16S library sizes for DA from non-SIBO and SIBO subjects.

| Library size  (sequences) | Non-SIBO  (N=98) | SIBO  (N=42) |
| --- | --- | --- |
| Mean | 212,202 | 203,098^a^ |
| Standard Deviation | 139,385 | 148,658 |
| Standard Error of Mean | 13,801 | 22,411 |
| Median | 183,761 | 172,713^a^ |
| 25% Percentile | 113,649 | 116,862 |
| 75% Percentile | 262,128 | 247,360 |

a – Mann-Whitney test P>0.05.
