# Supplementary material for: The duodenal microbiome is altered in small intestinal bacterial overgrowth
Source: PLoS One. 2020 Jul 9;15(7):e0234906. doi: 10.1371/journal.pone.0234906 (PMC7347122; doi:10.1371/journal.pone.0234906)
Supplement: S2 Table — (DOCX) [file pone.0234906.s004.docx]

**S2 Table.** Fold change in the relative abundance of Proteobacteria classes in DA from non-SIBO and SIBO subjects.

|  | Non-SIBO (N=98) vs. SIBO (N=42) | |
| --- | --- | --- |
| Taxonomy | Log 2 Fold Change | FDR P-value  Wald-test |
| Gammaproteobacteria | 4.73 | **<0.0001** |
| Deltaproteobacteria | 3.07 | **2.07E-7** |
| Alphaproteobacteria | -0.85 | 0.28 |
| Betaproteobacteria | -0.79 | 0.13 |
| Epsilonproteobacteria | -0.34 | 0.74 |

FDR P-value<0.05 are indicated in bold
