# Supplementary material for: The duodenal microbiome is altered in small intestinal bacterial overgrowth
Source: PLoS One. 2020 Jul 9;15(7):e0234906. doi: 10.1371/journal.pone.0234906 (PMC7347122; doi:10.1371/journal.pone.0234906)
Supplement: S3 Table — (DOCX) [file pone.0234906.s005.docx]

**S3 Table.** Picrust analysis KEGG 2 pathway of subjects with SIBO compared to non-SIBO subjects.

| **KEGG 2 level differences SIBO vs non-SIBO** | **Asymp. Sig. (2-tailed)** |
| --- | --- |
| Amino_Acid_Metabolism | 0.0008268218 |
| Biosynthesis_of_Other_Secondary_Metabolites | 0.0025856667 |
| Cancers | 0.0000060389 |
| Carbohydrate_Metabolism | 0.0003516242 |
| Cardiovascular_Diseases | 0.2662106188 |
| Cell_Communication | 1.0000000000 |
| Cell_Growth_and_Death | 0.9761834350 |
| Cell_Motility | 0.0000012943 |
| Cellular_Processes_and_Signaling | 0.0000004629 |
| Circulatory_System | 0.1904185095 |
| Digestive_System | 0.3936735566 |
| Endocrine_System | 0.0296239446 |
| Energy_Metabolism | 0.0002212972 |
| Environmental_Adaptation | 0.0000138706 |
| Enzyme_Families | 0.0000372144 |
| Excretory_System | 0.0090516066 |
| Folding_Sorting_and_Degradation | 0.0001115590 |
| Genetic_Information_Processing | 0.0000079951 |
| Glycan_Biosynthesis_and_Metabolism | 0.0000030747 |
| Immune_System | 0.0000068133 |
| Immune_System_Diseases | 0.0156896184 |
| Infectious_Diseases | 0.0000003056 |
| Lipid_Metabolism | 0.0000194364 |
| Membrane_Transport | 0.0000045457 |
| Metabolic_Diseases | 0.0179325333 |
| Metabolism | 0.0000020235 |
| Metabolism_of_Cofactors_and_Vitamins | 0.0015994784 |
| Metabolism_of_Other_Amino_Acids | 0.0000379100 |
| Metabolism_of_Terpenoids_and_Polyketides | 0.0165364449 |
| Nervous_System | 0.5031229019 |
| Neurodegenerative_Diseases | 0.0001196167 |
| Nucleotide_Metabolism | 0.3141709273 |
| Poorly_Characterized | 0.0000009777 |
| Replication_and_Repair | 0.1977494614 |
| Sensory_System | 1.0000000000 |
| Signal_Transduction | 0.0000012399 |
